# Mechanical simulation study of postoperative displacement of trochanteric fractures using the finite element method

**DOI:** 10.1186/s13018-018-1011-y

**Published:** 2018-11-27

**Authors:** Atsuo Furui, Nobuki Terada, Kazuaki Mito

**Affiliations:** Department of Orthopaedic Surgery Restorative Medicine of Neuro-Musculoskeletal System, Fujita Health University, Bantane Hospital, 3-6-10 Otoubashi, Nakagawa-ku, Nagoya, Aichi 454-8509 Japan

**Keywords:** Finite element method, Trochanteric fracture, Sliding hip screw, Angulation deformity, Postoperative displacement

## Abstract

**Background:**

Femoral trochanteric fractures are common among older adults. In the reduction of trochanteric fractures, acquiring the support of the anterior cortex at the fracture site on lateral view immediately after surgery is important. However, even if the cortical support is acquired, postoperative displacement due to the loss of this support often occurs. This study aimed to investigate local stress distribution in several trochanteric fracture models and to evaluate risk factors for postoperative displacement using the finite element (FE) method.

**Methods:**

Displaced two-fragment fracture models with an angulation deformity at the fracture site and a non-displaced two-fragment fracture model were constructed. The models with an angulation deformity were of two types, one with the proximal fragment directed backward (type A) and the other with the proximal fragment rotated forward from the femoral neck axis (type B). Thereafter, FE models of the femur and a sliding hip screw mounted on a 135° three-hole side-plate were constructed. A 2010-N load was applied to the femoral head, and a 1086-N load was applied to the greater trochanter. Under this condition, the maximum value of the von Mises stress distribution and the amount of displacement of the femoral head vertex in the distal direction were investigated.

**Results:**

A larger maximum stress value at the medial femoral neck cortex and a higher amount of displacement in the distal direction were particularly recognized in type A models. These results indicate that microstructural damage was larger in type A models and that type A fracture alignment may be particularly related to fracture collapse and subsequent postoperative displacement.

**Conclusion:**

Even if support of the anterior cortex at the fracture site on lateral view is acquired immediately after surgery, caution is necessary for cases in which the proximal fragment is directed backward in the postoperative displacement from the viewpoint of the biomechanics of the FE method.

## Background

Femoral trochanteric fractures are common among older adults. These fractures are closely related to osteoporosis due to aging and can become a serious public health challenge with the trend of population senility worldwide [1]. Early rigid fixation is necessary for femoral trochanteric fractures, and patients should participate in rehabilitation as soon as possible to reduce the complications associated with long-term immobilization [2].

A variety of implants have been used for internal fixation of femoral trochanteric fractures. Recently, plates and sliding screws have been the most commonly used implants for fixation and are currently the gold standard [3–5]. In our clinical cases, we used a sliding hip screw (SHS) mounted on a 135° three-hole side-plate device. This device was designed in such a way that impaction at the fracture site was controlled by the gliding screw [6], and stable contact at the fracture site was acquired during the postoperative period [7]. Controlled fracture impaction occurs when SHS contributes to axial and torsional stability in addition to sliding capability [8]. Several complications such as fracture collapse, failure of internal fixation, and non-union occur in cases wherein fracture reduction is suboptimal or is not maintained and the sliding effect is not functional [9].

**Fig. 1:**
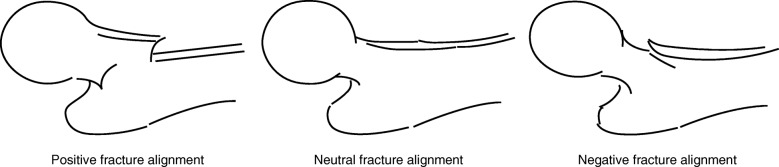
Classification of fracture alignments on the lateral view [11, 12]. Positive alignment, with the anteriorly displaced proximal fragment; neutral alignment, with continuous alignment between the proximal fragment and distal fragment; and negative alignment, with the posteriorly displaced proximal fragment are shown

**Fig. 2:**
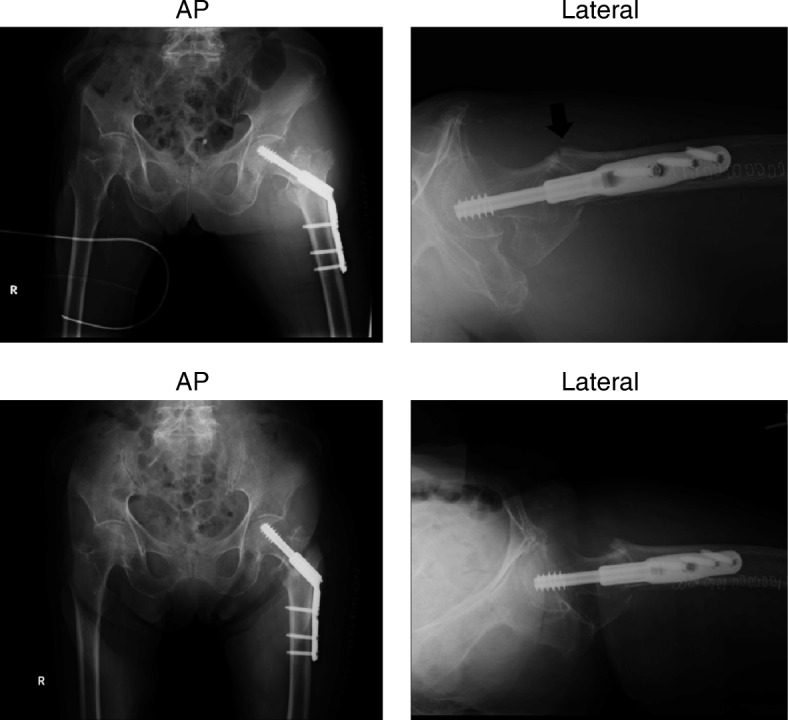
Postoperative displacement of trochanteric fractures. The upper section shows the postoperative radiograph, confirming reduction to the neutral fracture alignment. The lower section shows a radiograph, obtained on the postoperative day 14, confirming the negative fracture alignment. The black arrow indicates an angulation deformity at the fracture site

The purpose of reduction of trochanteric fractures is to acquire the support of the anterior cortex at the fracture site on lateral view [10]. The classification of fracture alignments on lateral view is shown in Fig. 1 [11, 12]. Reduction to a “positive” or “neutral” fracture alignment is necessary to acquire anterior cortical support. Reduction to the positive fracture alignment is ideal, as it provides sufficient support of the anterior femoral neck cortex. The most commonly encountered alignment is a reduction to the neutral fracture alignment, and it has been considered clinically acceptable, but postoperative displacement often occurs because of loss of anterior cortical support at the fracture site (i.e., displacement from the neutral fracture alignment to the negative fracture alignment). Several factors have been suggested to cause this postoperative displacement, and fracture alignment with an angulation deformity at the fracture site on lateral view is reported to be one of the main causes of displacement [11] (Fig. 2). We have postulated that unusual loading at the fracture site leads to fracture collapse, subsequent postoperative displacement, and cut-out of the lag screw (i.e., protrusion of the lag screw from the femoral head) in some cases. Therefore, this study aimed to investigate local stress distribution in models with an angulation deformity at the fracture site and to evaluate the risk factors for postoperative displacement using the finite element (FE) method.

## Methods

The study was approved by the ethics committee of Fujita Health University (No. HM18-078). The unaffected femur of a female patient (aged 95 years) was scanned using a computed tomography scanner (Brilliance 64, Philips Medical Systems, Cleveland, OH, USA). A series of images (slice thickness, 1.0 mm) of the femur were obtained. These images were imported in the Digital Imaging and Communications in Medicine format and were transferred to Mimics 18 (Materialise, Leuven, Belgium).

The fracture line was set from the upper part of the lesser trochanter to the greater trochanter and displaced two-fragment fracture models with an angulation deformity at the fracture site and a non-displaced two-fragment fracture model were simulated.

Models with an angulation deformity were of two types, one with the proximal fragment directed backward (type A) and the other with the proximal fragment rotated forward from the femoral neck axis (type B). Type A comprised three models in which the angulation angles at the fracture site were set at 10, 30, and 50°. Type B comprised three models in which the rotational angle of the proximal fragment was set at 10, 30, and 50°.

**Fig. 3:**
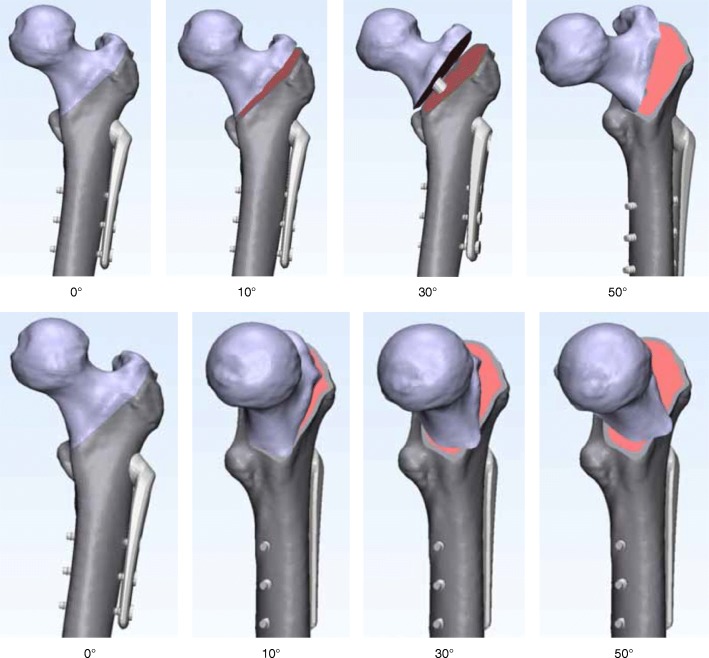
Models of the femur and a sliding hip screw. The upper section shows the non-displaced two-fragment fracture model and type A models. The lower section shows the non-displaced two-fragment fracture model and type B models

Models of the femur and an SHS mounted on a 135° three-hole side-plate were combined (Fig. 3). The geometrical dimensions of the SHS were obtained from the implant manufacturer’s catalog (Teijin Nakashima Medical Co., Ltd., Okayama, Japan).

Surface errors of the femoral bone models were corrected using 3-matic 11 software (Materialise). After correcting the surface roughness of the models, the models were converted to a primary tetrahedron mesh using HyperMesh software (Altair Engineering, Troy, MI, USA). The numbers of elements and nodes were 158.023 and 39.077, respectively. The FE models, as aforementioned, were imported into MSC Marc 2012 software (Shinjyuku, Tokyo, Japan) for FE analysis.

The material properties of the bone and SHS models were assumed to be linearly elastic and isotropic [13–16]. These were simplified models as the bone is nonlinear and anisotropic [17, 18]. The material of SHS was considered to be titanium. The modulus of elasticity was based on the study by Harrigan et al. [19] (Table 1).

**Table 1:** Material properties of femoral cortical and trabecular bones and the implant

	Young’s modulus (GPa)	Poisson’s ratio
Titanium (implant)	110	0.3
Cortical bone	16	0.3
Trabecular bone	1	0.3

Regarding friction coefficients, 0.1 was used for bone–bone interactions; 0.1, for bone–plate of the implant interactions [20]; 0.3, for bone–cortical screw of the implant interactions [21]; and 0.5, for implant–implant interactions.

The applied load condition was based on the study by Akay et al. [22]. A 2010-N load was applied to the femoral head, and a 1086-N load was applied to the greater trochanter. The distal section of the femoral shaft was fully restrained.

**Fig. 4:**
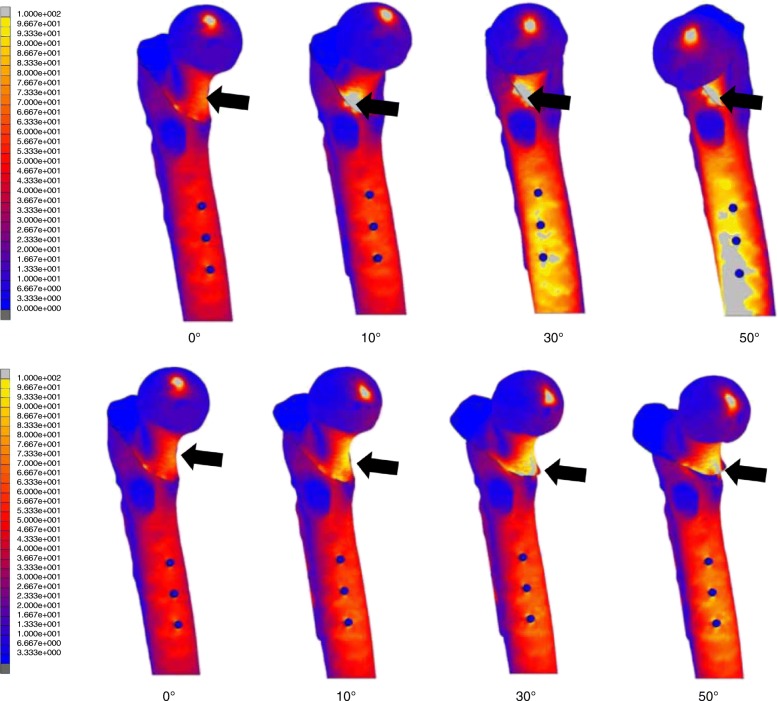
Maximum value view of the von Mises stress distribution (the medial femoral neck cortex). The upper section shows the non-displaced two-fragment fracture model and type A models. The lower section shows the non-displaced two-fragment fracture model and type B models. The black arrow indicates the maximum stress value at the medial femoral neck cortex

**Fig. 5:**
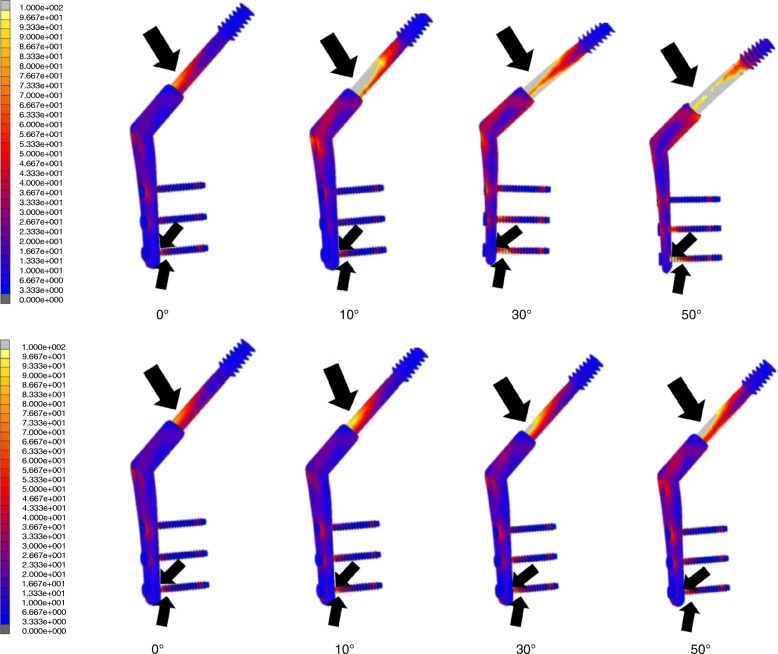
Maximum values of the von Mises stress distribution at SHS. The upper section shows the non-displaced two-fragment fracture model and type A models. The lower section shows the non-displaced two-fragment fracture model and type B models. The black arrow indicates the maximum stress values at the lag screw, the bottom of the third cortical screw, and the cortical screw–plate interface

In the simulation of these models, the maximum values of the von Mises stress distribution at the medial femoral neck cortex (Fig. 4) and the SHS (Fig. 5) were investigated.

The von Mises stress distribution is a common yield criterion used in the FE method [23]. The stress value corresponds to the equivalent stress, calculated from the principle stress in the three directions (*X*, *Y*, and *Z* axes) [24]. Furthermore, along with the amount of displacement in these three directions, the displacement of the femoral head vertex in the distal direction was also calculated.

## Results

### Maximum value of the von Mises stress distribution of the femur

In the simulation of all models, a large stress concentration of the von Mises stress distribution at the medial femoral neck cortex was observed. Compared with the non-displaced two-fragment fracture model, all type A models showed a much larger maximum value of the von Mises stress distribution at this site (Table 2). Furthermore, models with a larger angulation angle at the fracture site recognized a larger maximum stress value. In type B models, except the model with a rotational angle of 50°, the maximum stress value at the medial femoral neck cortex was smaller than that in the non-displaced two-fragment fracture model (Table 3).

**Table 2:** Maximum values of the von Mises stress distribution [MPa] in the non-displaced two-fragment fracture and type A models

	Medial femoral neck cortex	Lag screw	Cortical screw	Cortical screw–plate interface
	[Mpa]	[Mpa]	[Mpa]	[Mpa]
0°	147	260	314	131
10°	555	551	276	169
30°	620	777	540	230
50°	1031	1045	854	209

**Table 3:** Maximum values of the von Mises stress distribution [MPa] in the non-displaced two-fragment fracture and type B models

	Medial femoral neck cortex	Lag screw	Cortical screw	Cortical screw–plate interface
	[Mpa]	[Mpa]	[Mpa]	[Mpa]
0°	147	260	314	131
10°	109	313	426	196
30°	135	355	430	208
50°	187	454	400	192

### Maximum value of the von Mises stress distribution of the implant

In type A models, the maximum stress values at the lag screw, the bottom of the third cortical screw, and the cortical screw–plate interface were larger than those in the non-displaced two-fragment fracture model, except for the maximum stress value at the cortical screw in the model with an angulation angle of 10° (Table 2). In all type B models, the maximum stress values at the lag screw, the bottom of the third cortical screw, and the cortical screw–plate interface were a little larger than those in the non-displaced two-fragment fracture model (Table 3).

### Amount of displacement of the femoral head vertex in the distal direction

In all type A models, the femoral head vertex was displaced in the distal direction, and the amount of displacement was higher than that in the non-displaced two-fragment model (Table 4). In all type B models, the femoral head vertex was also displaced in the distal direction, and the amount of displacement was higher than that in the non-displaced two-fragment model (Table 5). However, the amount of displacement was more marked in type A models.

**Table 4:** Femoral head vertex displacement in the distal direction [mm] in the non-displaced two-fragment fracture and type A models

	0°	10°	30°	50°
	[mm]	[mm]	[mm]	[mm]
Displacement	1.79	2.98	5.77	8.21

**Table 5:** Femoral head vertex displacement in the distal direction [mm] in the non-displaced two-fragment fracture and type B models

	0°	10°	30°	50°
	[mm]	[mm]	[mm]	[mm]
Displacement	1.79	2.11	2.56	3.59

## Discussion

In the reduction of femoral trochanteric fractures, acquiring the support of the anterior cortex at the fracture site on lateral view immediately after surgery is important. However, even if anterior cortical support is acquired, postoperative displacement due to the loss of this support often occurs. Shiokawa et al. [25] reported a 26% incidence rate of this postoperative displacement, and we [11] observed a 30% incidence rate.

We [11] indicated that a fracture alignment with an angulation deformity at the fracture site on lateral view radiographs obtained immediately after surgery was a significant risk factor for postoperative displacement and that type A and B fracture alignments were distinguished on the basis of whether the fracture site angulation was easily reduced by pressure from the anterior direction during the intraoperative period. However, in this statistical analysis, fracture alignments with an angulation deformity did not include cases of type A but only included cases of type B. In this study, the relationship between this postoperative displacement and fracture alignments with an angulation deformity, including both type A and B deformities, was examined from the viewpoint of the biomechanics of the FE method.

The FE method has been widely used in biomechanics research. Many situations that would otherwise be difficult or impossible to study in practice can be simulated [13]. Currently, biomechanical analyses of trochanteric fractures and implants are commonly performed [9, 24, 26].

Stress distribution can be analyzed using the FE method by simulating the tissue of origin using discretized FE, setting parameters according to the actual material properties and load conditions, and calculating stress distribution using a computer [24]. If the stress is too large, the element will be damaged and the bone microstructure will be destroyed [24]. The von Mises stress distribution for implants is an indicator of the yielding of metals and can be explained by the mechanics of load sharing [15]. Higher stress value of implants leads to a higher possibility of implant failure [24]. Bone yielding contributes to internal fixation failure if the surrounding bone is weakened by yielding [27]. As a limit state, the failure occurs when elements of implants or the bone are exposed to a load, causing tensions that exceed the yield stress or strength [26]. In our simulation, other criteria such as minimum principal strain from strain analysis of the implant and bone were relatively large between the same types of models. In contrast, the von Mises stress distribution in the implant and bone showed a similar pattern in the same type of models. Therefore, the focus was on the von Mises stress distribution.

In our simulation, a large maximum value of the von Mises stress distribution at the medial femoral neck cortex and a high amount of displacement in the distal direction were especially observed in type A models. In type B models, the maximum stress values were smaller than those in the type A models. These results indicated that microstructural damage was larger in type A models than in type B models and that fracture alignment of type A may be particularly related to fracture collapse and subsequent postoperative displacement. We realized that these results may be due to insufficient bone contact between the proximal and distal fragments in type A, considering the wider bone contact in type B than in type A. Additionally, in type A, models with a larger angulation angle at the fracture site recognized a larger maximum stress value and a higher amount of displacement in the distal direction. These results may be because of an increase in insufficient bone contact between both fragments in models with a larger angulation angle, and the fracture alignment of these cases indicated an increasing risk of postoperative displacement.

Clinically, the fracture generally affects the bottom of the third cortical screw [24]. The yield strength of the medical titanium alloy is 850 to 900 MPa [28]. Therefore, the maximum stress values at SHS in all models, including both types A and B, except the model with an angulation angle of 50°, were almost in the safety range. In contrast, in the model with an angulation angle of 50°, the maximum stress values at the lag screw and the bottom of the third cortical screw were larger than the above yield strength. This may be because of significant bending of the lag screw due to consequential pressure. Therefore, in the fracture alignment of type A, cases with a striking increase in angulation angle should be warranted for implant failure.

In the FE analysis, preliminarily simulating every situation is impossible and this study has some limitations. In clinical practice, many cases include the medial fragment or the fractured greater trochanter, but we did not simulate fracture lines other than the anterior main fracture line. Furthermore, our mechanical simulation did not consider the factor of placement of the tip of the lag screw in the femoral head. Celik et al. [29] indicated that the position of the lag screw affects the risk of cut-out of the lag screw significantly and that the tip–apex distance [30], that is, the distance from the tip of the lag screw to the apex of the femoral head on anteroposterior and lateral radiographs, was a good predictor of cut-out risk using the FE method.

Another limitation of our FE analysis is that in our simulation, patient-specific measures of soft tissue thickness or muscle function were not considered. Konstantinidis et al. [31] concluded that muscle forces have little effect on fracture displacement in trochanteric fractures. However, these may be necessary for accurate results in our study. The other limitation is that bone quality (osteoporosis) was not assessed in our FE analysis. Further studies including these factors will be required.

## Conclusions

Our data indicate that mechanical simulation may be useful in the evaluation of factors related to postoperative displacement in trochanteric fractures and that our results can be applied in their clinical treatment. Even if reduction to the neutral fracture alignment is acquired immediately after surgery, caution is needed for cases in which the proximal fragment is directed backward in the postoperative displacement. Furthermore, with regard to fracture alignment, greater caution should be exercised for cases with a larger angulation angle at the fracture site in the postoperative displacement and implant failure.

## Abbreviations

FE: Finite element
SHS: Sliding hip screw
